# Allometric biomass equations for 12 tree species in coniferous and broadleaved mixed forests, Northeastern China

**DOI:** 10.1371/journal.pone.0186226

**Published:** 2018-01-19

**Authors:** Huaijiang He, Chunyu Zhang, Xiuhai Zhao, Folega Fousseni, Jinsong Wang, Haijun Dai, Song Yang, Qiang Zuo

**Affiliations:** 1 Key Laboratory for Forest Resources & Ecosystem Processes, Beijing Forestry University, Haidian District, Beijing, China; 2 Laboratoire de Botanique et d’Ecologie Vegetale, Universite de Lome, Lome-Togo; 3 Institute of Geographic Sciences and Natural Resources Research, Chinese Academy of Sciences, Chaoyang District, Beijing, China; Technical University in Zvolen, SLOVAKIA

## Abstract

Understanding forest carbon budget and dynamics for sustainable resource management and ecosystem functions requires quantification of above- and below-ground biomass at individual tree species and stand levels. In this study, a total of 122 trees (9–12 per species) were destructively sampled to determine above- and below-ground biomass of 12 tree species (*Acer mandshuricum*, *Acer mono*, *Betula platyphylla*, *Carpinus cordata*, *Fraxinus mandshurica*, *Juglans mandshurica*, *Maackia amurensis*, *P*. *koraiensis*, *Populus ussuriensis*, *Quercus mongolica*, *Tilia amurensis* and *Ulmus japonica*) in coniferous and broadleaved mixed forests of Northeastern China, an area of the largest natural forest in the country. Biomass allocation was examined and biomass models were developed using diameter as independent variable for individual tree species and all species combined. The results showed that the largest biomass allocation of all species combined was on stems (57.1%), followed by coarse root (21.3%), branch (18.7%), and foliage (2.9%). The log-transformed model was statistically significant for all biomass components, although predicting power was higher for species-specific models than for all species combined, general biomass models, and higher for stems, roots, above-ground biomass, and total tree biomass than for branch and foliage biomass. These findings supplement the previous studies on this forest type by additional sample trees, species and locations, and support biomass research on forest carbon budget and dynamics by management activities such as thinning and harvesting in the northeastern part of China.

## Introduction

Forests can accumulate a large amount of biomass and play an important role in regulating greenhouse gas emissions and maintaining atmospheric CO2 balance on earth[1]. About one third of the earth surface is covered by forests, of which China is one of the countries with abundant forest resource in world[2]. The contribution of forests to national carbon stock has been increasing in the last few decades, due to continued efforts of afforestation. According to the eighth national forest resource inventory (2008~2013), total area of forest has reached to 2.08×10^9^ ha, total growing stock to 1.51×10^11^ m^3^, and total forest cover to 21.4% [3]. The northeastern part of China has the largest reservoir of natural forests, representing 27.8% of the total area of forests and 27.5% of the total growing stock in the country [3]. The importance of quantifying biomass and carbon storage addresses the need to study relationships between growth and biomass components[4]. However, there are few studies which have adequately explored the relationships, especially in temperate coniferous and broad-leaved mixed forest in northeastern China[5–7].

Among the various methods available, allometric equations are the most common and reliable method for determining tree biomass[4] and carbon storage and flux[5,6] and a large number of allometric biomass equations have been developed for different forest tree species in many parts of the world[5,7–11]. Among the tree growth variables, diameter and height are most commonly used[11–14], due to their availability and easy to measure in forest inventories. Comparatively, diameter at breast height (DBH) can be more accurately measured and therefore, is relatively more reliable when using a single independent variable to develop biomass equation[5,7,8,11], although other growth variables such as tree height(H)[12,13,15,16], basal diameter (BD)[14,17], or even wood specific gravity (WSG)[5,18,19] are also used.

Relative to above-ground biomass (AGB) of tree stems, branches, and foliage, below-ground biomass (BGB) is harder to measure. While few studies have focused on determination BGB by developing equations based on easy to measure tree variables [9,20–23], it is still necessary for developing reliable BGB equations[24]. As a such, the root to shoot ratio (R:S) is commonly used to estimate BGB from AGB[2] in both in forest [1] and grassland [25] biomass studies.

In this study, we focused on 12 major tree species in the coniferous and broadleaved mixed forests, Northeastern China, *Pinus koraiensis*, *Quercus mongolica*, *Tilia amurensis*, *Fraxinus mandshurica*, *Juglans mandshurica*, *Acer mandshuricum*, *Acer mono*, *Ulmus japonica* and *Betula platyphylla* that dominate the upper layer and *Rhamnus davurica*, *Corylus mandshurica*, *Acer barbinerve*, *Carpinus cordata* and *Syringa reticulata* var. *Mandshurica that dominate* the lower canopy (see Table 1). Our objectives were: (1) to examine stand structures and species composition, (2) to develop allometric equations of individual species or general biomass equations for various biomass components (stems, branches, foliage and roots) using DBH, and (3) to investigate biomass allocation and above- and below-ground biomass relationships. Because the differences in environmental conditions caused by different study areas affect tree growth and biomass[22,26], we hope that this study will supplement these studies by Wang[22] and Cai et al[19].

**Table 1:** Species composition, density, DBH, H and basal area of living trees with DBH greater than 1cm in our study area.

Species	Density stems·ha^-1^	DBH(cm)		H(m)		Basal area,
		Mean±SD	Range	Mean±SD	Range	m^2^·ha^-1^
*A*. *mandshuricum*	67(5.53%)	6.62±7.48	1.2–43.2	5.67±3.17	1.5–18.0	0.52(1.83%)
*A*. *mono*	215(17.74%)	11.06±10.08	1.2–55.3	8.33±4.37	1.3–21.5	1.49(5.38%)
*B*. *platyphylla*	43(3.55%)	25.07±11.78	2.2–60.0	15.10±3.51	2.6–22.6	2.57(9.28%)
*C*. *cordata*	88(7.26%)	7.12±3.26	1.0–33.8	6.81±2.87	1.6–18.5	0.63(2.28%)
*F*. *mandshurica*	82(6.77%)	25.19±12.48	2.1–85.6	15.44±4.08	2.5–24.8	5.06(18.27%)
*J*. *mandshurica*	22(1.82%)	26.03±10.72	9.2–67.0	15.30±3.22	4.6–23.2	1.38(4.98%)
*M*. *amurensis*	25(2.06%)	11.53±6.55	1.0–40.1	9.24±3.31	1.9–17.6	0.34(1.23%)
*P*. *koraiensis*	98(8.09%)	14.67±13.10	1.4–63.2	8.55±4.71	1.7–22.8	2.97(10.73%)
*P*. *ussuriensis*	10(0.83%)	30.86±18.10	11.9–60.3	17.17±3.21	11.5–21.0	0.17(0.61%)
*Q*. *mongolica*	45(3.71%)	21.22±16.01	2.3–97.3	12.17±4.59	2.3–22.8	2.47(8.92%)
*T*. *amurensis*	125(10.31%)	15.20±11.65	1.4–77.3	10.47±4.54	2.0–23.8	3.59(12.96%)
*U*. *japonica*	157(12.95%)	12.71±11.59	1.3–81.4	8.72±5.15	1.3–22.8	3.65(13.18%)
Others	235(19.39%)	9.06±8.52	1.0–54.5	7.04±4.09	1.5–23.7	2.85(10.29%)
Total	1212	13.81±12.33	1.0–97.3	9.37±5.54	1.3–24.8	27.69

## Methods

### Ethics statement

All field studies were conducted in Jiaohe Forestry Experimental Bureau, who approved the permission for this research to conduct. We confirm that the field studies didn’t involve sampling of any endangered or protected species.

### Study site

The study was carried out in the Jiaohe Forestry Experimental Bureau(43°58′N, 127°43′E, elevation of 450 m a.s.l.), Jilin Province, Northeastern China. The climate is temperate continental, with a mean annual temperature of 3.8°C and a mean annual precipitation of 695.9 mm. The hottest month is July with a mean temperature of 21.7°C and the coldest month is January with the mean temperature of -18.6°C. The soil is a dark brown forest soil, and 20-100cm in depth [27].

**Fig 1:**
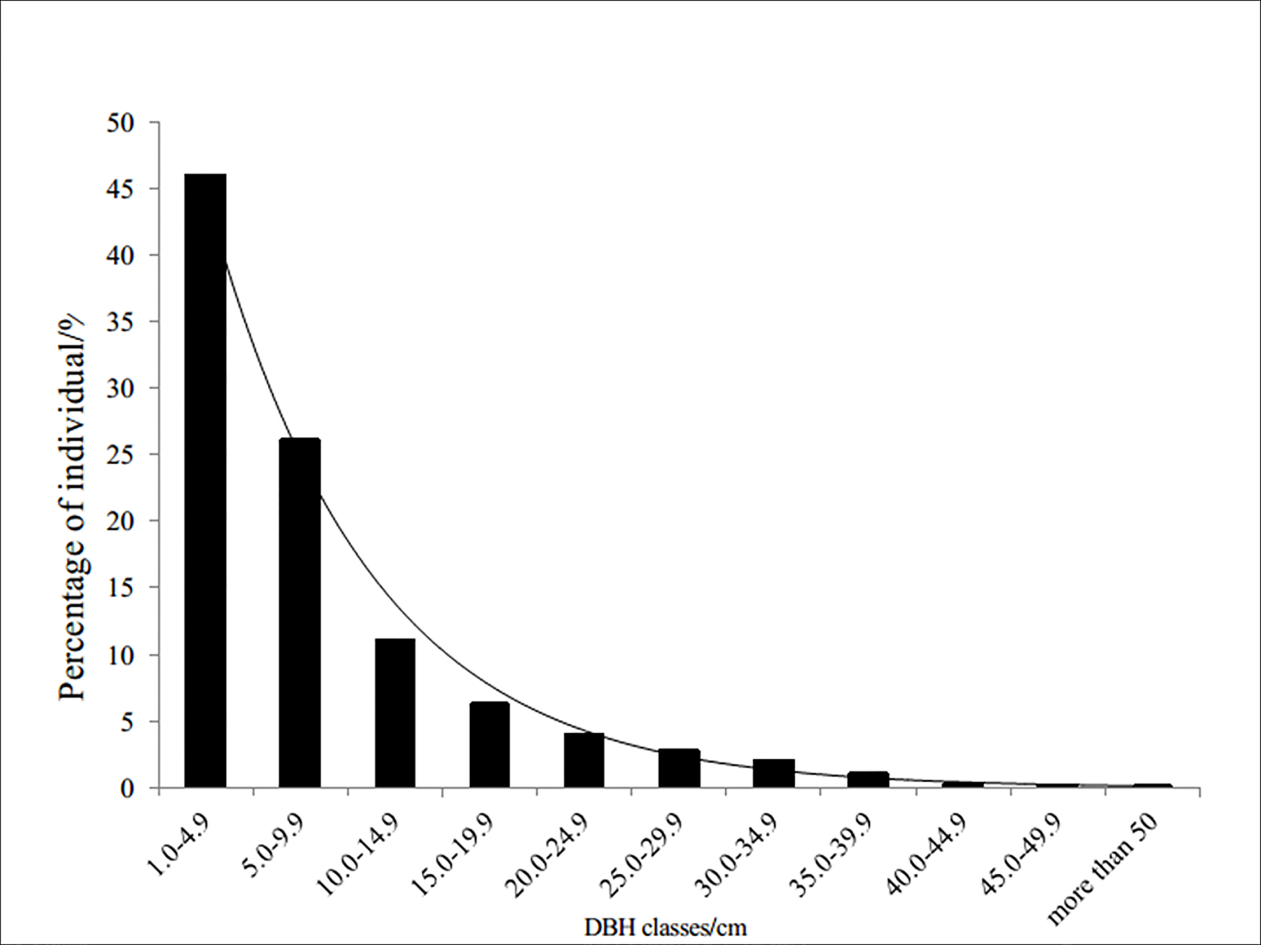
The DBH classes distribution of trees in our plots.

In 2011, four 100m × 100 m plots were established in the relatively homogeneous natural coniferous and broadleaf mixed stands. All trees with *DBH* ≥ 1 cm were measured for species name, DBH, tree height (H), and crown width (CW), tagged, and mapped for location. The characteristics of trees within the stands are shown in Table 1 and stand diameter distribution in Fig 1.

### Data collection

Destructive sampling in the field was conducted in July and August of 2012 when foliage biomass is the maximum[22]. A total of 122 healthy, defect-free trees were harvested, with 9–12 trees for each species (Table 2). After sample trees were felled at the ground surface, tree height (H), height to first live branch (H_1_), diameter at breast height (DBH), and diameter at the tree base (D_0_) were recorded. The crown length (CL) was defined as the difference between total tree height and height to the base of first live branch. Each tree crown was divided into three equal parts (upper layer, middle layer and lower layer). Within each layer, foliage was separated from branches, and both were weighed for total fresh weight. Stems were cut at 1.0 m, 1.3 m, 3.0 m and then at every 2 m above. The fresh weight of each stem section was recorded. A 5 cm thick disc was taken at the bottom of each stem section for moisture content determination in the laboratory. The moisture content of branches and foliage was determined from 500-1000g of fresh samples randomly selected within each layer. All branches and foliage of each layer were used for moisture content determination if their total weights were less than 1 kg.

**Table 2:** Descriptive statistics of the attributes measured on DBH and H of twelve sampled species.

species	N	DBH,cm		H,m	
		mean±SD	Range	mean±SD	Range
*Acer mandshuricum*	10	21.9±9.4b	7.8–35.9	15.6±2.9bc	9.1–18.5
*Acer mono*	12	24.4±12.2bc	6.4–45.3	16.3±4.0bcd	8.5–20.6
*Betula platyphylla*	10	22.8±11.3bc	5.7–40.0	19.0±4.5cd	9.3–22.8
*Carpinus cordata*	9	9.5±2.8a	5.1–13.4	10.1±1.2a	7.9–11.9
*Fraxinus mandshurica*	10	24.7±11.0bc	10.7–41.4	18.9±4.3cd	10.9–23.7
*Juglans mandshurica*	10	24.0±12.1bc	6.5–42.5	18.9±4.8cd	8.2–23.0
*Maackia amurensis*	10	13.7±6.8ab	4.9–25.4	13.2±3.7ab	7.0–18.2
*Pinus koraiensis*	11	24.8±12.5bc	8.4–44.0	14.8±5.2bc	6.7–22.3
*Populus ussuriensis*	10	27.0±12.9c	9.1–47.1	20.4±4.5d	10.5–26.4
*Quercus mongolica*	10	22.5±12.2bc	4.2–41.2	16.9±6.0bcd	5.5–22.8
*Tilia amurensis*	10	24.4±12.2bc	7.0–42.2	18.0±4.3cd	9.6–22.5
*Ulmus japonica*	10	22.7±11.6bc	5.6–39.9	16.2±4.2bc	6.8–20.1
Total	122	22.0±11.5	4.2–47.1	16.6±4.9	5.5–26.4

The value in the same column with different letters indicate a significant difference in twelve species (p<0.05). The lowercase and uppercase letters represent the components biomass and percent, respectively. N = number of sample trees for each species; SD = standard deviation.

Each sample tree was excavated for determination of root biomass. Because of high uncertainty and small proportion of fine roots in total root biomass, only coarse roots (diameter ≥ 5 mm) were counted[22]. The excavated roots were cleared of soil and foreign roots (roots from other plants), separated into stump and coarse roots, and weighed for fresh mass. About 500–1000 g fresh coarse roots and stump were chosen for each tree to determine moisture content (again, all coarse roots and stump were used if sample tree DBH was less than 10cm).

The stems, branches and root system of each sample tree were weighed with electronic platform balance (DCS-HT-A1, accuracy = 0.2kg), while the fresh weights of biomass samples for moisture content were determined with YP 30000 balance (accuracy = 1g). The biomass samples were dried at 85°C in the laboratory until a constant weight was reached. The dry weight of each biomass component was calculated with the dry/fresh weight ratio of biomass samples. Stem biomass was the total biomass of all stem sections, which, along with the sum of branches and foliage biomass in three crown layers, made above-ground biomass (AGB), while below-ground biomass (BGB) included biomass of stump and coarse roots. The biomass components of sample trees were summarized in S1 Table.

### Statistical analysis

We took the general biomass equation that has been widely used by others[8,28,29] to link diameter (X) with biomass components (Y) of each individual trees:

$$Y=aX^{b}$$ (1)

Because of the violation of heteroscedasticity assumption in nonlinear regression with original scales of measurements [30], the Eq (1) was log transformed:

$$\ln Y_{F}=a_{1}+b_{1}\ln X$$ (2)

$$\ln Y_{B}=a_{2}+b_{2}\ln X$$ (3)

$$\ln Y_{S}=a_{3}+b_{3}\ln X$$ (4)

$$\ln Y_{R}=a_{4}+b_{4}\ln X$$ (5)

The transformation, however, introduced a systematic bias, which can generally be corrected with the following correction factor (*CF*) [31]:

$$CF=\exp(SEE^{2}/2)$$ (6)

where *CF* is the correction factor, and *SEE* is the standard error of the estimate calculated as follows:

$$SEE=\sqrt{{\overset{n}{\underset{i=1}{\sum }}\left(\ln Y_{i}-ln{\hat{Y}}_{i}\right)}^{2}/(n-2)}$$ (7)

The Eqs (2) and (3) were back-transformed to get biomass equation[32]:

$$Y_{F}=e^{a_{1}}X^{b_{1}}CF_{1}$$ (8)

$$Y_{B}=e^{a_{2}}X^{b_{2}}CF_{2}$$ (9)

$$Ys=e^{a_{3}}X^{b_{3}}CF_{3}$$ (10)

$$Y_{R}=e^{a_{4}}X^{b_{4}}CF_{4}$$ (11)

The above-ground biomass was calculated by adding the foliage, branches and stems biomass. And the total biomass was calculated by adding the foliage, branches, stems and roots biomass.

$$\begin{array}{l} Y_{AGB}=Y_{F}+Y_{B}+Y_{S}\\ \;=e^{a_{1}}X^{b_{1}}CF_{1}+e^{a_{2}}X^{b_{2}}CF_{2}+e^{a_{3}}X^{b_{3}}CF_{3} \end{array}$$ (12)

$$\begin{array}{l} Y_{TB}=Y_{F}+Y_{B}+Y_{S}+Y_{R}\\ \;=e^{a_{1}}X^{b_{1}}CF_{1}+e^{a_{2}}X^{b_{2}}CF_{2}+e^{a_{3}}X^{b_{3}}CF_{3}+e^{a_{4}}X^{b_{4}}CF_{4} \end{array}$$ (13)

The goodness of fit of models was evaluated by the coefficients of determination (*R*^*2*^) and root mean square error (*RMSE*) calculated as follows:

$$RMSE=\sqrt{\sum_{i=1}^{n}\frac{{\left(\ln Y_{i}-\hat{\ln Y_{i}}\right)}^{2}}{n}}$$ (14)

Where *Y*_*i*_ and ${\hat{Y}}_{i}$ are observed and predicted biomass values of the *i*th sample tree, *n* is the number of sample trees, and *a*, *a*_1,_ *a*_2_, *a*_3_ and *a*_4_ is the scaling coefficient (or allometric constant) and *b*, *b*_1_,*b*_2_,*b*_3_ and *b*_4_ is the scaling exponent. The modeling was performed with R package *lm()* function and statistical comparisons were with R base package under R version 3.2.3.

The one Way-ANOVA was used to test the difference of above- and below-ground ratio among 12 species. The test was completed by SPSS 19.0 (SPSS, Inc, Chicago, IL) and the statistically different at p<0.05 level was significance.

## Results

### Stand characteristics

Stand density and basal area by species are presented in Table 1. *A*. *mono* was the most abundant species in density, accounting for 17.74% of the stand total trees, which is followed by *U*. *Japonica* (12.95%), *T*. *amurensis* (10.31%) and *P*. *koraiensis* (8.09%). The most abundant species by basal area is *F*. *mandshurica*, accounting for 18.28% of the stand total.

The DBH distribution of the studied stands followed a typical reversed J-shape curve (Fig 1) with the smallest diameter class (from 1.0 to 4.9 cm) accounting for 46.1% of the stand total and with the largest DBH class (≥ 50 cm) only for 0.1% of the stand total. The largest DBH (97.3 cm) and height (24.8 m) were in *Q*. *mongolica* and *F*. *mandshurica*, respectively. The largest average DBH and height were in *P*. *ussuriensis* (30.86cm and 17.17m, respectively) and the smallest were in *A*. *Mandshuricum* (6.62 cm and 5.67 m, respectively).

### Biomass allocation

**Fig 2:**
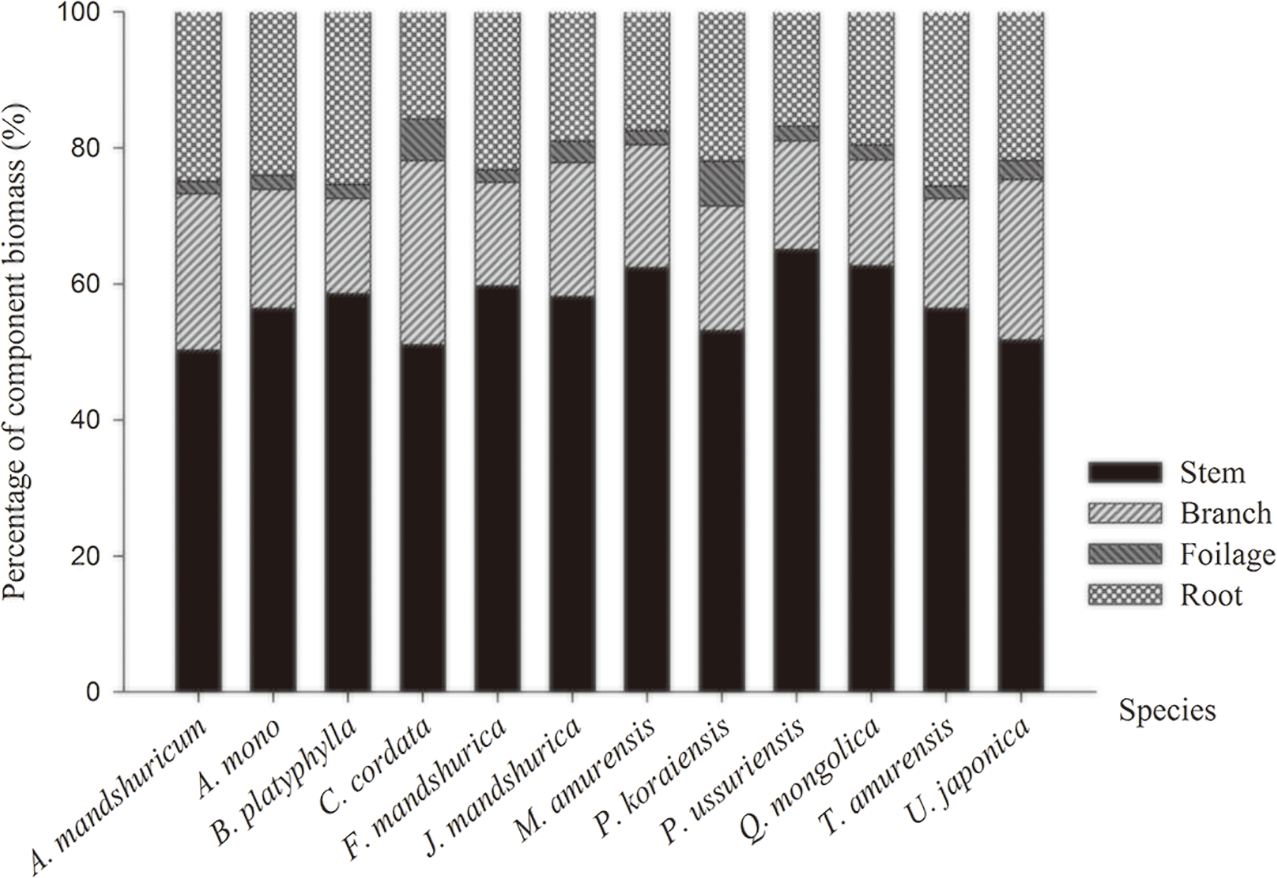
Average biomass percentage of stems, branches, foliage and roots of 122 trees individuals of 12 species.

Although total biomass and proportions of different biomass components varied among tree species, stems took the largest proportion of total tree biomass (57.1% on average), followed by roots (21.3%), branches (18.7%), and foliage (2.9%) (Table 3; Fig 2). Among the 12 species, *P*. *koraiensis* and *C*. *cordata* had the highest biomass allocation in foliage and *A*. *mandshuricum* and *F*. *mandshurica* had the lowest (*p*<0.05). The biomass allocation to branches was similar among the 12 species except for *C*. *cordata* and *B*. *platyphylla* (*C*. *cordata* was significantly higher than *B*. *platyphylla*). The largest stem allocation was in *P*. *ussuriensis* (65.0%) was the largest (65.0%) and the smallest in *A*. *mandshuricum* (50.2%). The biomass allocation to roots ranged from 15.6% to 25.8%, and was higher in *A*. *mandshuricum*, *A*. *mono*, *B*. *platyphylla* and *T*. *amurensis* than in *C*. *cordata*, *M*. *amurensis* and *P*. *ussuriensis*.

**Table 3:** The components biomass and proportion of twelve sample species.

Species	Items	Components biomass (kg) and proportion (%)					
		Foliage	Branches	Stem	Coarse root	AGB	Total
*Acer mandshuricum*	Mean±SD	5.3±4.7ab	118.4±134.2ab	185.5±148.3abc	92.9±73.5abc	309.2±278.8abc	402.1±350.3abc
	Range	1.0–16.2	2.0–399.0	12.1–415.5	7.3–415.5	15.1–815.4	22.7–1006.5
	Proportion±SD	1.8±1.2A	23.1±10.6BC	50.2±8.7A	24.9±4.1C	75.1±4.1A	100
*Acer mono*	Mean±SD	7.6±6.6ab	93.0±94.4ab	271.4±261.8c	120.5±120.6c	372.0±362.0bc	492.5±479.5bc
	Range	0.7–21.7	1.9–262.6	8.9–799.4	4.8–309.9	11.5–1083.7	16.3–1393.6
	Proportion±SD	2.0±0.9AB	17.5±6.5AB	56.4±6.1ABC	24.1±4.5C	75.9±4.5AB	100
*Betula platyphylla*	Mean±SD	9.0±8.0ab	85.1±93.5ab	235.0±201.7bc	134.1±136.6c	329.1±300.5abc	463.2±427.4bc
	Range	0.2–23.9	0.5–223.0	7.1–575.9	2.0–374.8	7.8–822.7	9.8–1197.5
	Proportion±SD	2.1±1.6AB	13.9±7.1A	58.2±11.2ABC	25.8±6.5C	74.2±6.5A	100
*Carpinus cordata*	Mean±SD	2.4±1.8ab	12.4±10.4a	19.0±11.5a	6.2±3.7a	33.7±23.2a	39.9±26.6a
	Range	0.5–6.4	1.1–33.2	5.0–38.6	0.9–11.1	6.6–78.2	7.6–89.3
	Proportion±SD	6.1±0.7C	27.1±8.9C	51.0±10.0A	15.8±3.7A	84.2±3.7D	100
*Fraxinus mandshurica*	Mean±SD	9.6±9.8ab	122.4±160.8ab	326.5±280.8c	149.3±157.5c	458.5±438.5c	607.8±594.0c
	Range	0.7–33.8	3.9–525.7	32.4–782.6	7.8–467.3	38.5–1342.2	46.4–1809.5
	Proportion±SD	1.8±1.9A	15.2±6.9AB	59.7±7.6ABC	23.3±4.2BC	76.8±4.2ABC	100
*Juglans mandshurica*	Mean±SD	10.4±10.7ab	114.5±148.3ab	214.3±179.0abc	84.9±86.8abc	339.3±331.2abc	424.2±417.6abc
	Range	0.9–32.0	1.8–442.9	6.0–489.6	2.8–242.3	10.3–964.5	13.1±1206.8
	Proportion±SD	3.1±1.0B	19.7±10.3ABC	58.1±12.2ABC	19.0±3.5AB	81.0±3.5CD	100
*Maackia amurensis*	Mean±SD	1.4±1.2a	26.0±39.7ab	54.7±53.6ab	17.5±20.1ab	82.1±92.5ab	99.6±112.4ab
	Range	0.2–3.4	0.8–122.7	4.2–150.4	1.3–61.2	5.2–276.5	6.5–337.6
	Proportion±SD	2.0±1.0A	18.2±10.4AB	62.4±8.7BC	17.5±4.0A	82.5±4.0CD	100
*Pinus koraiensis*	Mean±SD	22.8±22.0c	63.7±61.1ab	202.7±215.9abc	80.2±90.5abc	289.1±297.1abc	369.3±386.8abc
	Range	1.0–62.5	2.0–172.3	7.8–659.6	3.9–287.5	12.1–877.8	17.0–1165.3
	Proportion±SD	6.5±1.5C	18.3±3.7AB	53.1±5.6AB	22.0±3.2BC	78.0±3.2ABC	100
*Populus ussuriensis*	Mean±SD	7.7±7.7ab	83.9±95.0ab	255.2±237.9bc	69.4±65.5abc	346.8±337.1abc	416.2±400.9abc
	Range	0.4–25.9	2.6–261.9	13.0–711.0	3.4–189.8	17.4–983.9	20.8–1173.7
	Proportion±SD	2.2±1.1AB	16.0±5.8AB	65.0±6.6C	16.8±2.6A	83.2±2.6D	100
*Quercus mongolica*	Mean±SD	8.8±9.6ab	94.6±116.6ab	258.6±235.2bc	79.7±84.2abc	362.0±357.4bc	441.7±439.6abc
	Range	0.1–30.0	0.3–354.2	2.1–698.3	0.8–277.7	2.5–1082.5	3.3–1360.2
	Proportion±SD	2.2±0.8AB	15.6±9.3AB	62.6±9.6C	19.6±5.7BC	80.4±5.7BCD	100
*Tilia amurensis*	Mean±SD	7.1±7.1ab	80.4±85.2ab	211.6±196.1abc	93.8±79.8abc	299.1±284.8abc	392.9±363.1abc
	Range	0.3–20.2	1.0–251.9	6.8–550.5	2.8–235.2	8.0–769.1	10.8–1004.3
	Proportion±SD	1.8±0.5A	16.2±7.4AB	56.4±8.2ABC	25.6±5.2C	74.4±5.2A	100
*Ulmus japonica*	Mean±SD	12.9±13.7b	137.4±146.8b	209.0±194.5abc	108.2±103.7bc	359.3±348.8bc	467.5±448.6bc
	Range	0.4–42.1	1.6–410.1	5.4–612.3	1.4–298.2	7.5–1064.5	8.9–1362.7
	Proportion±SD	2.8±1.1AB	23.7±9.8BC	51.7±11.1A	21.8±5.2BC	78.2±5.2ABC	100
Total	Mean±SD	8.9±11.1	86.1±108.4	206.2±209.6	87.4±99.6	301.2±316.9	388.6±411.6
	Range	0.1–62.5	0.3–442.9	2.1–799.4	0.8–467.3	2.5–1342.2	3.3–1809.5
	Proportion±SD	2.9±1.9	18.7±8.7	57.1±9.6	21.3±5.5	78.7±5.6	100

The value in the same column with different letters indicate a significant difference in twelve species (p<0.05). The lowercase and uppercase letters represent the components biomass and percent, respectively. SD = standard deviation.

### Allometric biomass equations

**Fig 3:**
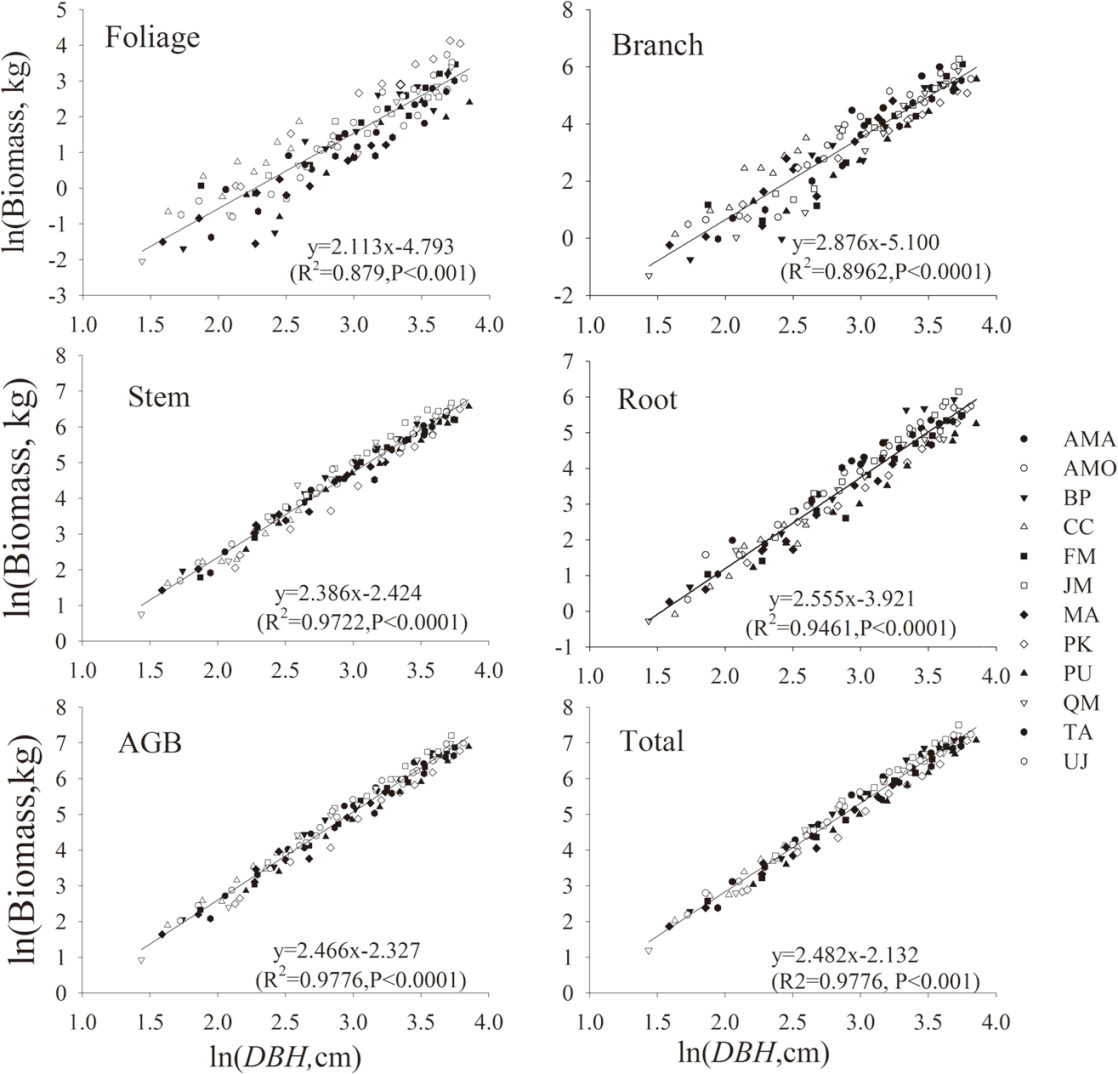
Linear regression equations of the natural log transformation of the biomass components of foliage, branch, stem, root, AGB and total from all tree species as a function of DBH (cm). AMA: *Acer mandshuricum*; AMO: *Acer mono*; BP: *Betula platyphylla*; CC: *Carpinus cordata*; FM: *Fraxinus mandshurica*; JM: *Juglans mandshurica*; MA: *Maackia amurensis*; PK: *Pinus koraiensis*; PU: *Populus ussuriensis*; QM: *Quercus mongolica*; TA: *Tilia amurensis*; UJ: *Ulmus japonica*.

The coefficients of log-transformed allometric biomass equations on DBH was significant for all species and biomass components (p<0.001, Fig 3, Table 4 and Table 5). In general, the allometric models were more accurate for individual species than for all species combined, and more robust for stem biomass, root biomass, above-ground biomass, and total biomass than for branch and foliage biomass. For example, the species-specific models explained more than 95% of the total variations, except for roots (*R*^2^ = 0.883) and stems (*R*^2^ = 0.942) in *C*. *cordata*. The biomass models for all species combined explained 97.8% of the total variation in total biomass, 97.2% in stem biomass, 94.6% in root biomass, 89.7% in branch biomass, and 83.7% in foliage biomass.

**Table 4:** Coefficients of allometric equations transformed as ln *Y_i_* = *a_i_* + *b_i_*ln*DBH* for 12 tree species for foliage, branch, stems and root. Where, when i = 1, the Y = Y_F_ = foliage biomass; when i = 2, the Y = Y_B_ = branch biomass; when i = 3, the Y = Y_S_ = stem biomass; when i = 4, the Y = Y_R_ = root biomass.

Species	Components	Coefficient		*R*^*2*^	*RMSE*	CF
		*a*_*i*_ (S.E.)	b_*i*_ (S.E.)			
*A*. *mandshuricum*	Foliage	-3.463 (0.772)**	1.606(0.255)***	0.832	0.293	1.070
	Branch	-6.005 (0.735)***	3.3230(0.243)***	0.959	0.312	1.063
	Stem	-2.111 (0.278)***	2.310(0.092)***	0.988	0.118	1.009
	Root	-2.786 (0.350)***	2.303(0.116)***	0.980	0.149	1.014
*A*. *mono*	Foliage	-3.948 (0.502)***	1.810(0.161)***	0.926	0.282	1.049
	Branch	-4.645 (0.856)***	2.740(0.275)***	0.908	0.482	1.150
	Stem	-2.164 (0.175)***	2.336(0.056)***	0.994	0.098	1.006
	Root	-3.098 (0.407)***	2.358(0.131)***	0.970	0.229	1.032
*B*. *platyphylla*	Foliage	-6.304 (1.089)***	2.599(0.3581)***	0.868	0.580	1.234
	Branch	-7.014 (1.131)***	3.445(0.372)***	0.915	0.603	1.255
	Stem	-1.941 (0.170)***	2.286(0.056)***	0.995	0.091	1.005
	Root	-4.354 (0.626)***	2.807(0.206)***	0.959	0.334	1.072
*C*. *cordata*	Foliage	-4.240 (0.855)**	2.200(0.384)***	0.824	0.304	1.061
	Branch	-5.416 (0.772)***	3.398(0.347)***	0.932	0.274	1.050
	Stem	-1.909 (0.440)**	2.111(0.197)***	0.942	0.156	1.016
	Root	-4.046 (0.781)**	2.544(0.351)***	0.883	0.278	1.051
*F*. *mandshurica*	Foliage	-5.454 (0.904)***	2.315(0.288)***	0.890	0.376	1.092
	Branch	-6.989 (0.875)***	3.481(0.279)***	0.951	0.363	1.086
	Stem	-2.301 (0.242)***	2.443(0.077)***	0.992	0.100	1.006
	Root	-4.360 (0.304)***	2.800(0.097)***	0.991	0.126	1.010
*J*. *mandshurica*	Foliage	-4.231 (0.616)***	1.974(0.200)***	0.924	0.328	1.070
	Branch	-5.768 (1.233)**	3.063(0.399)***	0.880	0.657	1.309
	Stem	-2.466 (0.280)***	2.381(0.091)***	0.989	0.149	1.014
	Root	-4.142 (0.507)***	2.565(0.164)***	0.968	0.270	1.047
*M*. *amurensis*	Foliage	-4.313 (0.733)***	1.700(0.288)***	0.813	0.409	1.110
	Branch	-5.524 (1.078)***	3.055(0.424)***	0.867	0.601	1.253
	Stem	-2.001 (0.256)***	2.198(0.101)***	0.984	0.143	1.013
	Root	-3.767 (0.357)***	2.391(0.140)***	0.973	0.199	1.025
*P*. *koraiensis*	Foliage	-5.179 (0.509)***	2.475(0.163)***	0.963	0.275	1.047
	Branch	-4.306 (0.393)***	2.527(0.126)***	0.978	0.212	1.028
	Stem	-3.394 (0.245)***	2.582(0.079)***	0.992	0.133	1.011
	Root	-3.779 (0.277)***	2.418(0.089)***	0.988	0.150	1.014
*P*. *ussuriensis*	Foliage	-5.506 (1.009)***	2.193(0.314)***	0.859	0.466	1.145
	Branch	-5.930 (0.618)***	2.975(0.192)***	0.968	0.286	1.052
	Stem	-2.507 (0.233)***	2.358(0.072)***	0.993	0.108	1.007
	Root	-4.208 (0.260)***	2.465(0.081)***	0.991	0.120	1.009
*Q*. *mongolica*	Foliage	-5.536 (0.355)***	2.346(0.118)***	0.980	0.229	1.033
	Branch	-6.503 (0.846)***	3.291(0.282)***	0.945	0.545	1.204
	Stem	-2.797 (0.386)***	2.571(0.128)***	0.980	0.248	1.039
	Root	-3.635 (0.302)***	2.452(0.101)***	0.987	0.195	1.024
*T*. *amurensis*	Foliage	-5.969 (0.600)***	2.368(0.193)***	0.949	0.313	1.063
	Branch	-6.171 (0.375)***	3.131(0.121)***	0.988	0.196	1.024
	Stem	-2.364 (0.391)***	2.323(0.126)***	0.977	0.204	1.026
	Root	-3.393 (0.501)***	2.398 (0.161)***	0.965	0.261	1.044
*U*. *japonica*	Foliage	-5.510 (0.597)***	2.438(0.198)***	0.956	0.339	1.076
	Branch	-5.056 (0.564)***	3.001(0.187)***	0.974	0.320	1.068
	Stem	-2.058 (0.339)***	2.271(0.112)***	0.983	0.192	1.024
	Root	-4.160 (0.358)***	2.690(0.118)***	0.987	0.203	1.027
all trees	Foliage	-4.793 (0.256)***	2.113 (0.085)***	0.837	0.562	1.174
	Branch	-5.100 (0.268)***	2.876 (0.090)***	0.896	0.591	1.194
	Stem	-2.424 (0.111)***	2.386 (0.037)***	0.972	0.243	1.031
	Root	-3.921 (0.167)***	2.555 (0.056)***	0.946	0.368	1.071

S.E. = standard error; *RMSE* = the root mean square error; *R*2 = the coefficient of determination and *CF* is a logarithmic correction factor; Root was defining as coarse roots (diameter more than 5mm).

**values are statistically different at 0.01 level of significance

*** value are statistically different at 0.001 level of significance.

**Table 5:** The equations of AGB and Total biomass of twelve species and all species.

Species	Components	Equations
*A*. *mandshuricum*	AGB	Y = 0.0335DBH^1.606^+0.0026DBH^3.323^+0.1222DBH^2.310^
	TB	Y = 0.0335DBH^1.606^+0.0026DBH^3.323^+0.1222DBH^2.310^+0.0625DBH^2.303^
*A*. *mono*	AGB	Y = 0.0202DBH^1.810^+0.0111DBH^2.740^+0.1156DBH^2.336^
	TB	Y = 0.0202DBH^1.810^+0.0111DBH^2.740^+0.1156DBH^2.336^+0.0466DBH^2.358^
*B*. *platyphylla*	AGB	Y = 0.0023DBH^2.599^+0.0011DBH^3.445^+0.1443DBH^2.286^
	TB	Y = 0.0023DBH^2.599^+0.0011DBH^3.445^+0.1443DBH^2.286^+0.0138DBH^2.807^
*C*. *cordata*	AGB	Y = 0.0153DBH^2.200^+0.0047DBH^3.398^+0.1506DBH^2.111^
	TB	Y = 0.0153DBH^2.200^+0.0047DBH^3.398^+0.1506DBH^2.111^+0.0184DBH^2.544^
*F*. *mandshurica*	AGB	Y = 0.0047DBH^2.315^+0.0010DBH^3.481^+0.1008DBH^2.443^
	TB	Y = 0.0047DBH^2.315^+0.0010DBH^3.481^+0.1008DBH^2.443^+0.0129DBH^2.800^
*J*. *mandshurica*	AGB	Y = 0.0156DBH^1.974^+0.0041DBH^3.063^+0.0861DBH^2.381^
	TB	Y = 0.0156DBH^1.974^+0.0041DBH^3.063^+0.0861DBH^2.381^+0.0166DBH^2.565^
*M*. *amurensis*	AGB	Y = 0.0149DBH^1.700^+0.0050DBH^3.055^+0.1370DBH^2.198^
	TB	Y = 0.0149DBH^1.700^+0.0050DBH^3.055^+0.1370DBH^2.198^+0.0237DBH^2.391^
*P*. *koraiensis*	AGB	Y = 0.0060DBH^2.475^+0.0139DBH^2.527^+0.0339DBH^2.582^
	TB	Y = 0.0060DBH^2.475^+0.0139DBH^2.527^+0.0339DBH^2.582^+0.0232DBH^2.418^
*P*. *ussuriensis*	AGB	Y = 0.0047DBH^2.193^+0.0028DBH^2.975^+0.0821DBH^2.358^
	TB	Y = 0.0047DBH^2.193^+0.0028DBH^2.975^+0.0821DBH^2.358^+0.0150DBH^2.465^
*Q*. *mongolica*	AGB	Y = 0.0041DBH^2.346^+0.0018DBH^3.291^+0.0634DBH^2.571^
	TB	Y = 0.0041DBH^2.346^+0.0018DBH^3.291^+0.0634DBH^2.571^+0.0270DBH^2.452^
*T*. *amurensis*	AGB	Y = 0.0027DBH^2.368^+0.0021DBH^3.131^+0.0965DBH^2.323^
	TB	Y = 0.0027DBH^2.368^+0.0021DBH^3.131^+0.0965DBH^2.323^+0.0351DBH^2.398^
*U*. *japonica*	AGB	Y = 0.0044DBH^2.438^+0.0068DBH^3.001^+0.1308DBH^2.271^
	TB	Y = 0.0044DBH^2.438^+0.0068DBH^3.001^+0.1308DBH^2.271^+0.0160DBH^2.690^
all	AGB	Y = 0.0097DBH^2.113^+0.0073DBH^2.876^+0.0913DBH^2.386^
	TB	Y = 0.0097DBH^2.113^+0.0073DBH^2.876^+0.0913DBH^2.386^+0.0212DBH^2.555^

### Above-and below-ground biomass relationships

**Fig 4:**
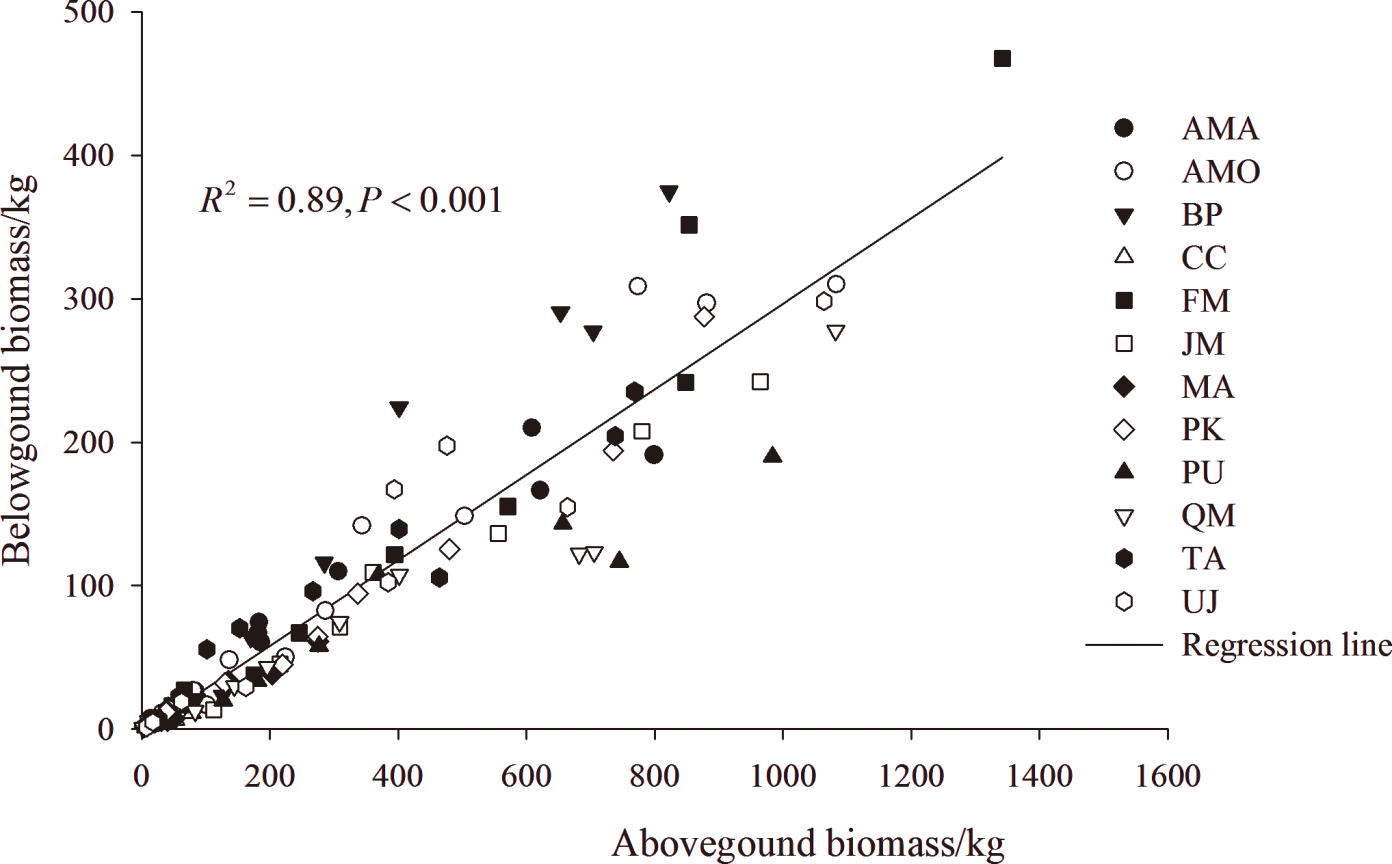
Above- and below-ground biomass relationships for 122 trees individual of 12 species. AMA: *Acer mandshuricum*; AMO: *Acer mono*; BP: *Betula platyphylla*; CC: *Carpinus cordata*; FM: *Fraxinus mandshurica*; JM: *Juglans mandshurica*; MA: *Maackia amurensis*; PK: *Pinus koraiensis*; PU: *Populus ussuriensis*; QM: *Quercus mongolica*; TA: *Tilia amurensis*; UJ: *Ulmus japonica*.

The below ground biomass (BGB) to above ground biomass (AGB) ratios ranged from 0.14 to 0.46 (average = 0.30) and significantly differed among the 12 species (*p* < 0.05. The lowest ratio was in *C*. *cordata* and the highest in *B*. *platyphylla* (Table 6). There was a significant linear relationship between AGB and BGB for individual species and all species combined (Fig 4 and Table 6). The coefficients of determination exceeded 0.9 for all species, except for *C*. *cordata* (R^2^ = 0.769).

**Table 6:** Coefficients of the linear equation *Y* = *aX* + *b* for twelve species about above- and below- ground biomass.

Species	*a* (S.E.)	*b* (S.E.)	*R*^2^
*A*. *mandshuricum*	0.259 (0.024)***	14.235 (9.753)	0.934
*A*. *mono*	0.327 (0.022)***	-0.968 (11.370)	0.955
*B*. *platyphylla*	0.457 (0.027)***	-10.146 (11.67)	0.973
*C*. *cordata*	0.141 (0.029)**	1.433 (1.174)	0.769
*F*. *mandshurica*	0.353 (0.024)***	-12.437 (14.731)	0.965
*J*. *mandshurica*	0.261 (0.010)***	-3.535 (4.469)	0.989
*M*. *amurensis*	0.214 (0.014)***	-0.110 (1.648)	0.968
*P*. *koraiensis*	0.301 (0.016)***	-6.778 (6.514)	0.975
*P*. *ussuriensis*	0.189 (0.017)***	4.054 (7.833)	0.947
*Q*. *mongolica*	0.229 (0.020)***	-2.988 (9.998)	0.941
*T*. *amurensis*	0.274 (0.021)***	11.962 (8.609)	0.9534
*U*. *japonica*	0.283 (0.035)***	6.540 (16.780)	0.906
All	0.298 (0.010)***	-1.711 (4.223)	0.888

**values are statistically different at 0.01 level of significance

*** value are statistically different at 0.001 level of significance.

## Discussion

The tree species we studied are commonly found in temperate coniferous and broadleaved mixed forests[33]. The reversed J-shape diameter distribution indicates a relative early stage of stand development, which helps explain lack of some shade tolerant conifers such as *Picea jezoensis*[22], *Picea koraiensis*, and *Abies nephrolepis*[19,34] that occur more at late successional stage of mature and over-mature stands.

Our findings on the biomass allocation among different parts of trees are consistent to the observations in temperate forests[34,35] and elsewhere with highest biomass allocation on stems and the lowest on foliage, while the ranking of biomass allocation on roots and branches varies among studies[23,36]. *P*. *ussuriensis* had the highest 65.0% allocation to stem biomass, likely due to their greater height and height to first live branch and therefore relatively smaller crown length and biomass allocation to branches and foliage biomass. Similarly, *A*. *mandshuricum* was relatively smaller in total height and the height to first live branch, resulting in proportionally small stem biomass (50.2%) and larger in branch and foliage biomass.

*P*. *koraiensis* was the only one coniferous tree species and had the highest foliage biomass allocation ratio among 12 species, and other studies also showed that the ratio of foliage biomass of coniferous species was generally higher than that of broadleaf species.[19,37–39]. This is likely due to evergreen nature of conifers that carry multi-year growth of foliage. Grote[38] studied foliage and branch biomass of six spruce and six beech species in Bavaria and shown that foliage biomass per unit area in spruce was almost three times greater than that in beech. In our study, *P*. *koraiensis*, *F*. *mandshurica*, *A*. *mono* and *T*. *amurensis* were similar in averages DBH (≈24 cm) and the average foliage biomass in *P*. *koraiensis* was about twice that in *F*. *mandshurica* and three times that in *A*. *mono* and *T*. *amurensis*.

As suggested by other studies[7,16,34,40–42], diameter is a reliable indicator for various biomass components of trees. Our findings are also along with those of others that stem, above-ground, roots and total biomass have less variations than branches and foliage and can be more accurately estimated with allometric equations in some tree species[35,36,43,44] such as *A*. *mandshuricum*, *C*. *cordata* and *J*. *mandshurica* in this study. This may have to do with the variation of local conditions, such as tree position in the canopy and light availability. The inclusion of tree height in diameter models may enhance model precision[13,19,22]; however, height may be more useful for stand biomass than for individual tree biomass according to the study by Wang et.al [45] in northeastern China.

As expected, all species combined, general biomass models have lower predicting power than species-specific models, consistent with the findings by others[5,46]. However, general biomass models can be an option when species-specific models are not available, particularly in estimation of large scale forest biomass. This approach can also be taken in estimation of below-ground biomass using BGB:AGB ratio [47], although the ratio differs with environmental (e.g., precipitation, soil moisture, soil texture and fertility)[48] and stand (such as stand age, height, forest type or forest origin) conditions[1,47,49], or even among different studies[2,22]. Again, species-specific ratio would be more accurate than all species combined, average BGB:AGB ratio (0.30 in this study), which was quite different from the estimates by Zhu et al.[33] (0.22) and Wang et al.[2] (0.39) in coniferous and broad-leaved mixed forest under similar climatic conditions of northeastern China. Other than the effects of environmental and stand conditions mentioned above, this difference may be largely due to the proportions of different tree species included, according to the species range of BGB:AGB ratio in this study (0.14 to 0.46).

## Conclusion

We examined biomass allocation including above- and below-ground biomass ratio and developed allometric equations for different biomass components of 12 individual tree species and all the species combined, in temperate coniferous and broadleaved mixed forests, northeastern China. Average biomass allocation was 57.1% on stems, 21.3% on roots, 18.7% on branches, and 2.9% on foliage, which varied among the species examined. Species-specific biomass allocation and allometric equations should be used for more accurate estimation; however, all species combined, general biomass allocation and allometric equations could provide good approximations when species-specific information is not available. Although models can be further refined by inclusion of more destructive samples and biomass allocation to roots can be slightly greater if fine roots are included, our results supplements the previous studies on this forest type by additional sample trees, species and locations, and would support biomass research on forest carbon budget and dynamics by management activities such as thinning and harvesting in the northeastern part of China.

## Supporting information

S1 Table Biomass data of 122 sample trees belonging to 12 species about foliage, branch, stem, coarse roots, AGB, BGB and TB. (DOC)
