# Supplementary material for: Allometric biomass equations for 12 tree species in coniferous and broadleaved mixed forests, Northeastern China
Source: PLoS One. 2018 Jan 19;13(1):e0186226. doi: 10.1371/journal.pone.0186226 (PMC5774681; doi:10.1371/journal.pone.0186226)
Supplement: S1 Table — (DOC) [file pone.0186226.s001.doc]

**S1 Table. Biomass data of 122 sample trees belonging to 12 species about foliage, branch, stem, coarse roots, AGB, BGB and TB.**

| Species | DBH,cm | H,m | Foliage,kg | Branch,kg | Stem,kg | Root,kg | AGB,kg | BGB,kg | TB,kg |
| --- | --- | --- | --- | --- | --- | --- | --- | --- | --- |
| AMA | 7.8 | 9.1 | 0.962 | 2.011 | 12.125 | 7.266 | 15.098 | 7.266 | 22.364 |
| AMA | 12.4 | 12.7 | 2.481 | 12.042 | 41.100 | 16.424 | 55.623 | 16.424 | 72.048 |
| AMA | 14.7 | 15.8 | 1.702 | 15.384 | 68.460 | 26.320 | 85.546 | 26.320 | 111.866 |
| AMA | 18.8 | 14.2 | 4.585 | 87.570 | 94.028 | 66.981 | 186.183 | 66.981 | 253.163 |
| AMA | 20.1 | 17 | 2.342 | 37.176 | 148.872 | 60.620 | 188.389 | 60.620 | 249.009 |
| AMA | 20.6 | 16 | 3.182 | 51.238 | 132.304 | 74.421 | 186.725 | 74.421 | 261.146 |
| AMA | 23.7 | 18.5 | 4.769 | 95.542 | 211.461 | 109.896 | 311.771 | 109.896 | 421.667 |
| AMA | 31.5 | 17.3 | 10.373 | 290.857 | 330.969 | 166.176 | 632.199 | 166.176 | 798.375 |
| AMA | 33.8 | 17.8 | 6.131 | 193.624 | 415.494 | 209.885 | 615.248 | 209.885 | 825.133 |
| AMA | 35.9 | 18 | 16.202 | 399.011 | 400.172 | 191.112 | 815.384 | 191.112 | 1006.496 |
| AMO | 6.4 | 8.5 | 0.698 | 1.904 | 8.879 | 4.846 | 11.482 | 4.846 | 16.327 |
| AMO | 10.9 | 11.4 | 0.836 | 2.083 | 29.682 | 11.180 | 32.601 | 11.180 | 43.781 |
| AMO | 15.3 | 13.5 | 3.008 | 16.015 | 62.249 | 26.970 | 81.272 | 26.970 | 108.242 |
| AMO | 15.7 | 15.8 | 2.899 | 25.832 | 73.243 | 16.777 | 101.973 | 16.777 | 118.750 |
| AMO | 17.8 | 14.3 | 3.174 | 52.870 | 81.180 | 48.232 | 137.223 | 48.232 | 185.455 |
| AMO | 20 | 18.5 | 6.301 | 70.576 | 147.475 | 50.029 | 224.352 | 50.029 | 274.380 |
| AMO | 23.8 | 19.5 | 8.957 | 47.638 | 230.377 | 82.554 | 286.972 | 82.554 | 369.526 |
| AMO | 29 | 17 | 5.713 | 66.488 | 271.761 | 141.829 | 343.962 | 141.829 | 485.791 |
| AMO | 32.1 | 18.7 | 7.635 | 115.737 | 380.937 | 148.325 | 504.310 | 148.325 | 652.635 |
| AMO | 36.9 | 20.6 | 14.446 | 208.115 | 551.716 | 308.510 | 774.276 | 308.510 | 1082.786 |
| AMO | 40 | 20.6 | 15.695 | 246.605 | 619.386 | 296.755 | 881.686 | 296.755 | 1178.440 |
| AMO | 45.3 | 19.6 | 21.670 | 262.612 | 799.428 | 309.938 | 1083.710 | 309.938 | 1393.647 |
| BP | 5.7 | 9.3 | 0.185 | 0.479 | 7.131 | 1.971 | 7.795 | 1.971 | 9.766 |
| BP | 11.2 | 13.8 | 0.287 | 0.978 | 32.942 | 8.790 | 34.207 | 8.790 | 42.997 |
| BP | 14 | 17 | 3.752 | 18.605 | 62.526 | 20.827 | 84.883 | 20.827 | 105.710 |
| BP | 16.3 | 20.5 | 3.042 | 25.990 | 97.761 | 23.177 | 126.793 | 23.177 | 149.969 |
| BP | 20.4 | 18.7 | 4.935 | 15.299 | 150.281 | 63.961 | 170.516 | 63.961 | 234.477 |
| BP | 24 | 21.7 | 13.637 | 50.170 | 221.075 | 116.040 | 284.882 | 116.040 | 400.923 |
| BP | 28.1 | 22.4 | 14.230 | 96.906 | 290.443 | 280.012 | 401.579 | 280.012 | 681.591 |
| BP | 32.1 | 22.6 | 17.184 | 196.387 | 439.711 | 290.499 | 653.282 | 290.499 | 943.781 |
| BP | 36.1 | 21.4 | 8.848 | 222.980 | 472.438 | 161.298 | 704.266 | 161.298 | 865.564 |
| BP | 40 | 22.8 | 23.939 | 222.808 | 575.931 | 374.796 | 822.677 | 374.796 | 1197.473 |
| CC | 5.1 | 7.9 | 0.515 | 1.143 | 4.987 | 0.912 | 6.645 | 0.912 | 7.557 |
| CC | 6.6 | 9.1 | 1.385 | 2.613 | 9.154 | 1.967 | 13.151 | 1.967 | 15.118 |
| CC | 7.6 | 9.8 | 0.785 | 2.893 | 9.246 | 2.630 | 12.924 | 2.630 | 15.554 |
| CC | 8.5 | 9.9 | 2.083 | 11.519 | 9.747 | 6.125 | 23.350 | 6.125 | 29.475 |
| CC | 9.6 | 11.1 | 1.564 | 11.589 | 21.118 | 7.319 | 34.270 | 7.319 | 41.589 |
| CC | 10.5 | 10.6 | 2.008 | 9.627 | 20.161 | 7.833 | 31.796 | 7.833 | 39.629 |
| CC | 11.4 | 11.9 | 3.601 | 17.726 | 28.435 | 11.137 | 49.762 | 11.137 | 60.899 |
| CC | 12.6 | 11.2 | 3.001 | 21.144 | 29.177 | 6.549 | 53.323 | 6.549 | 59.872 |
| CC | 13.4 | 9.4 | 6.416 | 33.163 | 38.587 | 11.124 | 78.166 | 11.124 | 89.290 |
| FM | 6.5 | 8.2 | 1.073 | 3.226 | 5.954 | 2.818 | 10.253 | 2.818 | 13.071 |
| FM | 9.7 | 12.9 | 0.864 | 1.841 | 18.090 | 4.080 | 20.795 | 4.080 | 24.875 |
| FM | 14.5 | 18.6 | 1.907 | 3.130 | 56.592 | 16.685 | 61.630 | 16.685 | 78.315 |
| FM | 18 | 19.7 | 4.134 | 14.157 | 94.201 | 13.545 | 112.492 | 13.545 | 126.037 |
| FM | 21.2 | 19.5 | 6.273 | 59.850 | 149.676 | 45.380 | 215.798 | 45.380 | 261.178 |
| FM | 25.8 | 22.5 | 9.279 | 72.547 | 227.551 | 71.104 | 309.378 | 71.104 | 380.482 |
| FM | 30.1 | 21.7 | 7.572 | 71.590 | 282.338 | 109.265 | 361.499 | 109.265 | 470.764 |
| FM | 34.1 | 22 | 16.670 | 185.931 | 354.076 | 136.503 | 556.677 | 136.503 | 693.180 |
| FM | 37.8 | 21.4 | 24.680 | 290.277 | 465.008 | 207.549 | 779.965 | 207.549 | 987.514 |
| FM | 42.5 | 23 | 32.012 | 442.912 | 489.574 | 242.279 | 964.497 | 242.279 | 1206.777 |
| JM | 10.7 | 14.2 | 1.420 | 4.740 | 32.359 | 7.839 | 38.518 | 7.839 | 46.358 |
| JM | 12.2 | 10.9 | 0.743 | 3.852 | 42.652 | 16.348 | 47.247 | 16.348 | 63.595 |
| JM | 14.2 | 14 | 1.803 | 5.644 | 59.391 | 27.054 | 66.838 | 27.054 | 93.892 |
| JM | 17.5 | 19.4 | 6.461 | 43.744 | 125.019 | 37.477 | 175.223 | 37.477 | 212.700 |
| JM | 22.2 | 20.1 | 4.624 | 47.975 | 193.590 | 67.062 | 246.189 | 67.062 | 313.251 |
| JM | 26.5 | 20.5 | 8.046 | 76.524 | 310.198 | 121.804 | 394.768 | 121.804 | 516.571 |
| JM | 29.5 | 21.1 | 13.573 | 105.368 | 452.345 | 155.178 | 571.287 | 155.178 | 726.464 |
| JM | 34.7 | 23.7 | 12.607 | 188.719 | 647.440 | 241.526 | 848.766 | 241.526 | 1090.292 |
| JM | 37.6 | 22.8 | 12.852 | 221.916 | 619.535 | 351.519 | 854.303 | 351.519 | 1205.822 |
| JM | 41.4 | 21.9 | 33.802 | 525.738 | 782.610 | 467.302 | 1342.151 | 467.302 | 1809.452 |
| MA | 4.9 | 7 | 0.223 | 0.788 | 4.150 | 1.304 | 5.160 | 1.304 | 6.464 |
| MA | 6.4 | 8.3 | 0.431 | 1.063 | 7.526 | 1.841 | 9.020 | 1.841 | 10.860 |
| MA | 9.7 | 12.5 | 0.212 | 1.542 | 20.659 | 5.431 | 22.413 | 5.431 | 27.843 |
| MA | 9.8 | 13 | 0.881 | 5.102 | 25.825 | 5.637 | 31.808 | 5.637 | 37.445 |
| MA | 11.6 | 14 | 1.289 | 16.221 | 34.878 | 7.076 | 52.388 | 7.076 | 59.464 |
| MA | 12.2 | 11.2 | 0.823 | 11.177 | 29.273 | 5.621 | 41.273 | 5.621 | 46.894 |
| MA | 14.5 | 13.2 | 1.058 | 4.323 | 37.493 | 14.868 | 42.874 | 14.868 | 57.742 |
| MA | 19.2 | 18.2 | 2.165 | 29.429 | 104.404 | 33.711 | 135.999 | 33.711 | 169.710 |
| MA | 22.8 | 16.5 | 3.309 | 68.103 | 132.645 | 38.284 | 204.057 | 38.284 | 242.342 |
| MA | 25.4 | 17.5 | 3.359 | 122.667 | 150.439 | 61.159 | 276.465 | 61.159 | 337.624 |
| PK | 8.4 | 6.7 | 1.070 | 3.271 | 7.809 | 4.844 | 12.151 | 4.844 | 16.995 |
| PK | 8.7 | 8.6 | 1.043 | 2.011 | 11.101 | 3.882 | 14.155 | 3.882 | 18.037 |
| PK | 12.6 | 10.3 | 4.630 | 11.658 | 22.837 | 12.119 | 39.126 | 12.119 | 51.245 |
| PK | 17 | 11 | 3.351 | 16.391 | 38.348 | 19.032 | 58.089 | 19.032 | 77.121 |
| PK | 20.8 | 12.3 | 14.382 | 38.896 | 77.110 | 31.760 | 130.388 | 31.760 | 162.149 |
| PK | 24.7 | 17.1 | 18.597 | 42.926 | 158.771 | 44.767 | 220.294 | 44.767 | 265.061 |
| PK | 28.3 | 16.9 | 18.215 | 62.501 | 194.181 | 64.145 | 274.897 | 64.145 | 339.042 |
| PK | 31.6 | 18.5 | 32.168 | 75.489 | 229.943 | 94.445 | 337.600 | 94.445 | 432.045 |
| PK | 36 | 18.1 | 37.305 | 114.061 | 328.910 | 125.204 | 480.276 | 125.204 | 605.479 |
| PK | 40.8 | 20.9 | 62.532 | 172.291 | 500.890 | 194.273 | 735.713 | 194.273 | 929.985 |
| PK | 44 | 22.3 | 57.297 | 160.918 | 659.616 | 287.467 | 877.831 | 287.467 | 1165.298 |
| PU | 9.1 | 10.5 | 0.822 | 3.647 | 12.958 | 3.390 | 17.426 | 3.390 | 20.816 |
| PU | 11.6 | 15.7 | 0.449 | 2.562 | 26.809 | 6.727 | 29.820 | 6.727 | 36.547 |
| PU | 16.4 | 19.6 | 1.509 | 8.928 | 68.619 | 15.841 | 79.056 | 15.841 | 94.897 |
| PU | 19.9 | 20.7 | 2.430 | 15.143 | 109.894 | 20.023 | 127.467 | 20.023 | 147.490 |
| PU | 24.4 | 20.3 | 6.222 | 31.926 | 142.923 | 33.619 | 181.071 | 33.619 | 214.689 |
| PU | 28.3 | 23 | 9.631 | 51.758 | 215.771 | 57.939 | 277.160 | 57.939 | 335.099 |
| PU | 33.1 | 22.3 | 11.483 | 84.209 | 274.055 | 107.302 | 369.746 | 107.302 | 477.047 |
| PU | 39.6 | 24.4 | 7.267 | 191.378 | 546.435 | 116.595 | 745.080 | 116.595 | 861.675 |
| PU | 40.3 | 20.7 | 25.917 | 187.761 | 443.574 | 143.067 | 657.251 | 143.067 | 800.318 |
| PU | 47.1 | 26.4 | 11.066 | 261.866 | 710.975 | 189.817 | 983.907 | 189.817 | 1173.724 |
| QM | 4.2 | 5.5 | 0.130 | 0.272 | 2.123 | 0.762 | 2.525 | 0.762 | 3.287 |
| QM | 8 | 8.4 | 0.474 | 1.037 | 9.425 | 5.462 | 10.935 | 5.462 | 16.397 |
| QM | 13.3 | 17.4 | 1.907 | 2.474 | 78.867 | 12.504 | 83.249 | 12.504 | 95.753 |
| QM | 17 | 16 | 3.431 | 47.791 | 93.419 | 30.055 | 144.641 | 30.055 | 174.696 |
| QM | 20.7 | 20.5 | 2.629 | 21.516 | 172.083 | 43.197 | 196.228 | 43.197 | 239.425 |
| QM | 23.7 | 19.8 | 6.076 | 39.727 | 262.910 | 74.598 | 308.712 | 74.598 | 383.310 |
| QM | 27.5 | 19.5 | 11.266 | 105.326 | 285.614 | 107.345 | 402.205 | 107.345 | 509.550 |
| QM | 32.2 | 21.1 | 15.165 | 161.501 | 506.213 | 122.034 | 682.880 | 122.034 | 804.914 |
| QM | 37 | 20.8 | 17.415 | 211.664 | 477.049 | 123.475 | 706.128 | 123.475 | 829.603 |
| QM | 41.2 | 22.8 | 30.001 | 354.240 | 698.267 | 277.675 | 1082.508 | 277.675 | 1360.183 |
| TA | 7 | 9.6 | 0.253 | 0.976 | 6.772 | 2.825 | 8.002 | 2.825 | 10.827 |
| TA | 9.9 | 13 | 0.525 | 2.711 | 24.169 | 6.571 | 27.404 | 6.571 | 33.975 |
| TA | 14 | 16.4 | 1.937 | 7.426 | 49.002 | 22.710 | 58.366 | 22.710 | 81.075 |
| TA | 17.5 | 17.5 | 2.460 | 12.669 | 86.848 | 55.548 | 101.977 | 55.548 | 157.525 |
| TA | 23.5 | 16 | 2.483 | 58.825 | 91.613 | 70.442 | 152.921 | 70.442 | 223.362 |
| TA | 26.7 | 20.5 | 4.159 | 50.524 | 213.080 | 96.014 | 267.762 | 96.014 | 363.777 |
| TA | 29.5 | 20.7 | 13.331 | 114.689 | 274.008 | 139.450 | 402.028 | 139.450 | 541.477 |
| TA | 33.9 | 22.5 | 10.659 | 131.754 | 322.492 | 105.457 | 464.905 | 105.457 | 570.362 |
| TA | 39.7 | 22.2 | 15.021 | 172.949 | 550.513 | 204.185 | 738.483 | 204.185 | 942.668 |
| TA | 42.2 | 21.3 | 20.232 | 251.851 | 497.020 | 235.205 | 769.102 | 235.205 | 1004.307 |
| UJ | 5.6 | 6.8 | 0.476 | 1.633 | 5.440 | 1.386 | 7.548 | 1.386 | 8.934 |
| UJ | 8.2 | 11.7 | 0.449 | 2.170 | 15.181 | 4.821 | 17.799 | 4.821 | 22.620 |
| UJ | 13.5 | 15.4 | 1.340 | 12.953 | 47.799 | 19.114 | 62.091 | 19.114 | 81.205 |
| UJ | 17.2 | 14.6 | 4.299 | 35.147 | 123.164 | 29.153 | 162.610 | 29.153 | 191.763 |
| UJ | 21.2 | 16.3 | 7.472 | 65.020 | 134.787 | 60.752 | 207.279 | 60.752 | 268.031 |
| UJ | 24.8 | 18.8 | 14.784 | 172.419 | 197.207 | 102.033 | 384.410 | 102.033 | 486.443 |
| UJ | 28.8 | 17.9 | 13.125 | 152.769 | 228.643 | 166.962 | 394.537 | 166.962 | 561.499 |
| UJ | 31.2 | 18.9 | 16.074 | 128.192 | 331.616 | 197.614 | 475.882 | 197.614 | 673.496 |
| UJ | 36.1 | 19.2 | 23.674 | 321.379 | 319.313 | 154.730 | 664.366 | 154.730 | 819.096 |
| UJ | 39.9 | 20.1 | 42.104 | 410.083 | 612.294 | 298.184 | 1064.481 | 298.184 | 1362.664 |

**Where, roots is defined as coarse roots; BGB=coarse roots biomass; AGB=foliage biomass+branch biomass+stem biomass and TB=BGB+AGB.** AMA: *Acer mandshuricum*; AMO: *Acer mono*; BP: *Betula platyphylla*; CC: *Carpinus cordata*; FM: *Fraxinus mandshurica*; JM: *Juglans mandshurica*; MA: *Maackia amurensis*; PK: *Pinus koraiensis*; PU: *Populus ussuriensis*; QM: *Quercus mongolica*; TA: *Tilia amurensis*; UJ: *Ulmus japonica* .
